# Unveiling the roles of *CaSDH8* in *Candida albicans*: Implications for virulence and azole resistance

**DOI:** 10.1080/21505594.2024.2405000

**Published:** 2024-10-15

**Authors:** Mingjiao Huang, Dongxu Song, Luoxiong Zhou, Zhenlong Jiao, Longbing Yang, Yang Yang, Jian Peng, Guo Guo

**Affiliations:** a School of Basic Medical Sciences, Guizhou Key Laboratory of Microbio and Infectious Disease Prevention & Control, Guizhou Medical University, Guiyang, China; b Key Laboratory of Environmental Pollution Monitoring and Disease Control, Guizhou Medical University, Ministry of Education, Guiyang, China; c Translational Medicine Research Center, Guizhou Medical University, Guiyang, China

**Keywords:** *Candida albicans*, CRISPR-Cas9, *SDH8*, mitochondria, azole resistance, virulence

## Abstract

*Candida albicans* is the most common pathogen in systemic fungal diseases, exhibits a complex pathogenic mechanism, and is increasingly becoming drug tolerant. Therefore, it is particularly important to study the genes associated with virulence and resistance of *C. albicans*. Here, we identified a gene (*orf19.1588*) that encodes a conserved mitochondrial protein known as *CaSDH8*, upon deletion of *CaSdh8*, the deleted strain (*Casdh8Δ/Δ*) experienced impaired growth, hyphal development, and virulence. *Casdh8Δ/Δ* displayed a reduced capacity to utilize alternative carbon sources, along with detrimental alterations in reactive oxygen species (ROS), mitochondrial membrane potential (MMP) depolarization, and adenosine triphosphate (ATP) levels. Interestingly, *Casdh8Δ/Δ* demonstrated resistance to azole drugs, and under the influence of fluconazole, the cell membrane permeability and mitochondrial function of *Casdh8Δ/Δ* were less compromised than those of the wild type, indicating a reduction in the detrimental effects of fluconazole on *Casdh8Δ/Δ*. These findings highlight the significance of *CaSDH8* as a crucial gene for the maintenance of cellular homoeostasis. Our study is the first to document the effects of the *CaSDH8* gene on the virulence and azole resistance of *C. albicans* at both the molecular and animal levels, providing new clues and directions for the antifungal infection and the discovery of antifungal drug targets.

## Introduction

On 25 October 2022, the World Health Organization (WHO) published a list of pathogen fungal priorities that classifies fungal pathogens into three categories based on their priority: critical priority group, high priority group, and medium priority group. *Candida albicans* belongs to the critical priority fungal pathogen, and the infection of *C.albicans* has been ranked first among invasive fungal infection in humans [1]. Therefore, studying the pathogenic and drug resistance mechanisms of *C. albicans* infection is particularly important.

At present, most researchers agree that *C. albicans* pathogenicity to the host largely depends on the fungi’s virulence factors and their interactions with the host [2]. Research on fungal virulence factors has predominantly focused on the secretion of hydrolases, metabolism, morphological transformations, cell adhesion and invasion, and biofilm formation. These virulence factors enable fungi to sense and adapt to diverse microenvironments within the host, ensuring their survival under host pressure and facilitating the effective initiation of infection [3–5]. During long-term co-evolution with humans, *C. albicans* has developed the ability to escape or disrupt the host’s antimicrobial defence response, in which morphological plasticity is a key determinant [6]. In addition, resistance to antifungal drugs in *C. albicans* involves multiple factors, such as changes in cell membrane and cell wall components, overexpression of efflux pump genes, mutations in target proteins, and biofilm formation are well defined [7]. Despite the continuous development of new antifungal drugs, safe and effective antifungal drugs for the clinical treatment of *C. albicans* infections remain limited to polyenes (Amphotericin B and nystatin), echinocandins (caspofungin, micafungin, and anidulafungin), and triazoles (fluconazole and ketoconazole, itraconazole, etc.) [8]. Among them, triazoles are widely used in clinical practice because of their strong oral bioavailability, extensive antifungal spectrum, and low toxic side effects [9]. However, azole resistance is a major factor contributing to clinical treatment failure [10] given the persistent preventative use and extended drug therapy cycles in patients with candidiasis. Therefore, it is crucial to study the azole resistance of *C. albicans* as soon as possible, and novel resistance genes and mechanisms remain to be discovered.

In this study, we discovered a gene (*orf19.1588*) encoding a conserved mitochondrial protein called *CaSdh8*, which was up-regulated in response to treatment with the antimicrobial peptide AMP-17 in *C. albicans* [11]. The gene is predicted to be located in the mitochondria and encodes a protein called succinate dehydrogenase assembly factor 4 (SDHAF4), which is homologous to Sdh8p in *Saccharomyces cerevisiae*. The succinate dehydrogenase complex (SDH) has four subunits (Sdh1/2/3/4) that play major roles in *S. cerevisiae*. Sdh5/6/7/8 were subsequently identified as assembly factors that assist the assembly of the four subunits into SDH, with *SDH8* ranked 8th according to the order of discovery after the four subunits, and since it is ranked 4th among the assembly factors, its full name is SDHAF4 [12]. SDH, also known as mitochondrial complex II, plays a crucial role in cellular energy production and is one of the hubs linking the tricarboxylic acid cycle (TCA) with oxidative phosphorylation, functioning as the sole mitochondrial inner membrane protein in the TCA [13]. It is also an important enzyme in the execution of healthy mitochondrial processes, and its deficiency can lead to various diseases [14]. Jonathan G et al. discovered a novel SDHAF known as Sdh8p, which is highly conserved across evolutionary time in mammals and *S. cerevisiae* [15]. Sdh8p interacts with the catalytic subunit Sdh1p in the mitochondrial matrix, facilitating its binding to Sdh2p and the subsequent assembly of the complete SDH complex, which is necessary to sustain succinate dehydrogenase (SDH) assembly and its enzymatic activity. The prevention of motor abnormalities, neurodegeneration, and accumulation of excess reactive oxygen species (ROS) in *Drosophila* requires the effects of Sdh8p [15]. However, the function of *SDH8* in *Candida albicans* remains unclear.

Here, we report the characterization of *SDH8* in *C. albicans*. Deletion of *SDH8* in *C. albicans* leads to inhibition of hyphal development, reduced virulence in mouse models of systemic candidiasis, and decreased sensitivity to azole drugs. Importantly, we also investigated the potential mechanisms of the *CaSDH8* gene in the virulence and azole drug sensitivity of *C. albicans*, offering a new avenue for the development of antifungal drug targets.

## Materials and methods

### Strains and plasmids

The *Candida albicans* SC5314 used in this study was donated by Dr Zhenbo Xu (South China University of Technology). The pADH99 and pADH100 plasmids were purchased from Hernday Laboratory [16]. DH5α was purchased from Takara (Shiga, Japan). The pET-28a-γmGFP plasmid was synthesized by Shanghai Sangon Biotechnology. 23–1359 and 23–3192 are clinical azole-resistant strains of *Candida albicans* and were collected from the Clinical Laboratory of the Affiliated Hospital of Guizhou Medical University. The other fungal strains were constructed in our laboratory. These strains were stored at −80°C and revitalized in YPD broth (Solarbio, Beijing, China) at 30°C with 16 ~ 18 h shaking (200 rpm). The genotype of each strain is listed in Table 1.

**Table 1.** *Candida albicans* strains used in this study.

Strains	Genotype	Parent strain	Reference
SC5314	Wild type		
*Casdh8Δ/Δ*	*Casdh8Δ/sdh8Δ*	SC5314	This study
CP	*Casdh8Δ/sdh8Δ::SDH8*	*Casdh8Δ/Δ*	This study
*Casdh8-*γm*GFP*	*Casdh8Δ/sdh8Δ::SDH8-*γm*GFP*	*Casdh8Δ/Δ*	This study
*Casdh8*^OE^	*Casdh8Δ/sdh8Δ::ENO1pro-SDH8*	*Casdh8Δ/Δ*	This study
23-1359	Clinical strain		
23-3192	Clinical strain		

### Gene deletion and complementation of CaSDH8

The target gene was knocked out using the CRISPR/Cas9 gene editing technique developed by Nguyen et al. [16]. A 20 bp guide RNA (gRNA) target sequence was designed using the online software Benchling (www.benchling.com) with the “Design and Analyze Guides” tool. Subsequently, the following flanking sequence was added to both ends of the designed gRNA: 5”-CGTAAACTATTTTTAATTTG (gRNA) GTTTTAGAGCTAGAAATAGC-3,” which was sent to Shanghai Sangon for synthesis. The pADH100-gRNA expression cassette was constructed by inserting the designed 20 bp gRNA into the successfully linearized pADH100 expression plasmid using Circular Polymerase Extension Cloning (CPEC). Using the Overlap-PCR method, a DNA sequence approximately 500 bp upstream and downstream of the target gene was selected as the repair template, and forward and reverse primers were designed on both sides of the sequence to amplify a donor DNA band of approximately 1000 bp by ligation with the ADD-TAG sequence. The plasmids pADH99 and pADH100-gRNA were digested with the FastDigest MssI enzyme, and the linearized plasmids and donor DNA were transfected into SC5314 competent cells. Transformants were screened on YPD media containing 200 μg/mL NAT.

For complementary strain, SC5314 was amplified using the flanking-specific primers of the target gene. A 500 bp upstream and downstream of the target gene and its CDS region was used as the repair homologous flanks, a pADH100 expression plasmid targeting 20 bp ADD-TAG sequence was inserted as complementary plasmid and transfected into competent cells of the knockout strain together with the pADH99 plasmid. Transformants were screened on YPD media containing 200 μg/mL NAT.

Single colonies with successfully deleted and complementary target genes were induced with YPM to produce FLIP recombinase, thereby removing the labels of CRISPR components and NATs.

### γmGFP fluorescent tagging and overexpression of CaSDH8 [17–19]

To determine the localization of the *SDH8* in *C. albicans*, we inserted the codon-optimized γmGFP fluorescent tags after the *SDH8* gene. SC5314 was used as a template to amplify the 500 bp fragments upstream and downstream of *SDH8* and *SDH8* gene, and then the γmGFP fragment was amplified using pET28a-γmGFP as a template. The four fragments were purified and ligated using Gibson Assembly Mix (Abclonal, China), then integrated into the Xhol1 and Bamh1 double digested plasmid pET28α (vector: insert = 1:1). A fusion fragment (Donor DNA) was amplified in the recombinant plasmid and transformed into SC5314 receptor cells using the SDH8-gRNA recognition sequence. Genetic transformation and subsequent screening of the fungi were performed as previously described. After successful construction of *Casdh8-*γm*GFP* strain, it was activated to the logarithmic growth stage. After washing, the concentration of fungal cells was adjusted to 2.0 × 10^6^ CFU/mL with sterile PBS. The mitochondrial tracker red fluorescent probe (TMR, MitoScene^TM^ Red CMXRos, Beyotime) with a final concentration of 50–200 nM was mixed with it and cultured at 37°C for 10 ~ 15 min. After washing the excess dye, it was fixed with 4% paraformaldehyde for 10 min, washed with PBS for 3 times, centrifuge to remove supernatant, resuspended the fungal cells with 10 μL of sterile PBS, and then the suspension was mixed with anti-fluorescent quenching agent and dropped on the slide. The subcellular localization of *SDH8* was observed by CLSM.

For the overexpression of *CaSdh8*, the ENO1 promoter sequence was utilized as the promoter for *CaSDH8*. The donor DNA construct was assembled by connecting the ENO1 promoter fragment, *CaSDH8* gene fragment, and the 500 bp fragments upstream and downstream of *CaSDH8* through overlay PCR. Similarly, a gRNA targeting *CaSDH8* was incorporated into the pADH100 vector. Genetic transformation and fungal screening were performed following the same procedure described earlier.

### Assessment of phenotypic properties

#### Determination of the liquid growth curve

The fungal cells in medium with glucose or acetate as carbon sources, respectively, were adjusted to a concentration of 2.0 × 10^6^ CFU/mL using a Neubauer-improved Counting Chamber, and then cultured at 30°C for 24 h. At 2-h intervals, 200 µL of each suspension was sampled, and the absorbance values were measured at OD_630_ nm using an iMark^TM^ microplate reader (BioRad, United States).

#### Hyphal formation assay

The fungal suspension was carefully adjusted to a concentration of 2.0 × 10^6^ CFU/mL using PBS buffer to ensure consistent cell density. Subsequently, 10 μL of each suspension was inoculated onto solid media, including YPD + 10% FBS and Spider media. The plates were then incubated at 37°C for 5 d to allow colony growth. Photos were taken periodically to observe and document colony morphology. Alternatively, to induce hyphal development, the activated fungal suspension was adjusted to a concentration of 2.0 × 10^6^ CFU/mL using liquid hyphal induction media, such as YPD + 10% FBS and Spider. Next, 1 mL of fungal cell suspension was added to the wells of a 24-well microtiter plate, and incubated at 37°C for 1.5 or 3 h to allow for hyphal development. Hyphal growth and morphology were observed and documented for further analysis using a Nikon Eclipse Ts2 inverted fluorescent microscope (Nikon, Japan).

#### Biofilm growth assay

The XTT (Beyotime, China) reduction assay was used to detect biofilm metabolic activity. A 200 μL of *C. albicans* cells at a concentration of 2 × 10^6^ CFU/mL was added to the wells of a 96-well microtiter plate, then incubated in YPD + 10%FBS or Spider medium for 24 to allow biofilm formation. After gently washing the biofilm, 150 µL of the XTT/menadione mixture was added to each well and incubated at 37°C for 3 h in the dark, then the OD_490_ was detected by iMark^TM^ microplate reader. To observe the biofilm, *C. albicans* cells were pre-grown in YPD + 10%FBS or Spider at 37°C for 24, then washed three times and incubated with 500 μL of propidium iodide (PI, 10 μg/mL, Sigma, United States) and SYTO9 (10 mm, Invitrogen, United States) for 15 min at 37°C in the dark. Following another round of PBS washing, the cell-attached slides inside the well plates were carefully clipped using sterile tweezers and immediately inverted onto sterile glass slides. Images were captured using a SpinSR10 Confocal Laser Scanning Microscopy (CLSM, Olympus Corporation, Japan) with excitation wavelengths of 488 nm for SYTO9 and 525 nm for PI.

#### Determination of drug susceptibility

Drug susceptibility was determined using the micro-liquid dilution method, following the guidelines recommended by the American Clinical and Laboratory Standards Institute (CLSI) M38-A2 [20] and M27-A3 [21]. *C. albicans* cells in the logarithmic growth phase were collected and resuspended in YPD broth, and the concentration was adjusted to 2.0 × 10^3^ CFU/mL. For the drug susceptibility test, 100 µL of the drug and 100 µL of cell suspension were inoculated into individual wells of a 96-well plate. The plates were incubated at 30°C for 48 h. The growth of fungal cells was assessed by measuring the optical density (OD_630_ nm) of each well using an iMark^TM^ microplate reader, and the results were supplemented by visual inspection. The minimum inhibitory concentration (MIC) of the tested drug was determined as the lowest concentration in the wells where no visible growth was observed, compared to the negative control. This assay was repeated thrice, with three technical replicates each time.

#### Spot assay

*C. albicans* cells in the logarithmic growth phase were collected, and the concentration of the fungal solution was adjusted to 1.0 × 10^7^ CFU/mL with YPD liquid medium, and then the cell suspension was diluted to 5 concentration gradients with sterile 3 µL of PBS. A fungal suspension was dripped onto a drug-containing YPD solid plate. The plates were dried, inverted, and cultured in a 30°C incubator for 48 h. The growth status of the colonies was observed and photographed.

### Mitochondrial function-related assays

#### Carbon source utilization

The monoclonal strains were activated by incubation in YPD liquid medium. After activation, the strains were washed with PBS and resuspended in order to adjust the concentration to 1.0 × 10^8^ CFU/mL. Subsequently, 5 μl of the suspension was dropped onto YP-X (Dextrose, Maltose, Sucrose, Glycerol, ethanol, and Sodium Acetate) solid media for streak inoculation. The plates were then incubated at 30°C for 24 h. The growth of each strain was carefully observed on both fermentable and non-fermentable carbon sources.

#### Reactive oxygen species (ROS), mitochondrial membrane potential (MMP) and adenosine triphosphate (ATP) measurement

*C. albicans* cells in the logarithmic growth phase in 5 mL YP-A (acetate) liquid media were collected by centrifugation and washed twice with sterile PBS buffer, and the concentration of the fungal suspension was adjusted with 1 mL PBS to 2.0 × 10^6^ CFU/mL. Alternatively, fungal cells in the logarithmic growth phase in 5 mL YP-D (glucose) liquid media were collected and were added 1 mL YPD liquid media containing different concentrations of FLC (2 µg/mL, 8 µg/mL, 16 µg/mL), then the concentration was adjusted to 2.0 × 10^6^ CFU/mL. After incubating with shaking for 8 h (30°C, 200 rpm), the concentration of fungal suspension was adjusted with 1 mL PBS to 2.0 × 10^6^ CFU/mL again. The fungal cells treated in two different ways were used for the subsequent detection of ROS, MMP and ATP.

Intracellular ROS production was detected by staining cells with DCFH-DA (Sigma, United States) dye. DCFH-DA with a final concentration of 10 μM was added to the treated fungal suspension (2.0 × 10^6^ CFU/mL) and incubated for 20 min at 30°C in the dark. After washing with sterile PBS to remove the excess dye, the supernatant was removed by centrifugation. Then, 50 μL of PBS was added to resuspension the fungal precipitation, and 10 μL of the resuspension was pipetted onto a slide. After mixing the anti-fluorescence quenching agent, the cells were covered with a coverslip and photographed using an Olympus CLSM instrument. For the quantitative analysis of ROS, the cells were incubated and treated following the same procedure described above. The samples were then detected by a multifunctional enzyme marker (BioTek, USA) with excitation/emission wavelengths of 485/530 nm.

MMP levels were measured using the mitochondrial membrane potential assay kit with JC-1 (Shanghai Beyotime, China). A 500 μL JC-1 staining solution was added to 500 μL treated fungal suspension (2.0 × 10^6^ CFU/mL), and the mixture was incubated for 20 min at 37°C. After incubation, the supernatant was removed by centrifugation at 600 g for 5 min at 4°C, then the cells were washed twice with JC-1 staining buffer and resuspended with 500 μL of JC-1 buffer, follwed by detecting on the machine immediately. The CytoFLEX LX flow cytometry (Beckman Coulter, United States) was used for single-cell fluorescence detection, and 10,000 cells were recorded in each sample. The emitted green fluorescence was measured at 530 nm, whereas the red fluorescence was measured at 590 nm.

Intracellular ATP content was measured using the ATP Assay Kit (Shanghai Beyotime, China). The prepared sample cells were lysed by adding lysate at a ratio of 200 μL lysate for each well of the 6-well plate, and it can be boiled for 1–2 min to completely lyse all cells within the suspension. The supernatant was centrifuged (4°C 12,000 rpm for 5 min) and placed in an ice box for subsequent determination. For ATP detection, 100 μL of ATP working solution was added to the wells and incubated at room temperature for 3–5 min to consume background ATP. Subsequently, 100 μL of sample was added to the wells and mixed rapidly. After a minimum interval of 2 s, relative light unit (RLU) values were measured using a multifunctional enzyme marker (BioTek, USA). The ATP concentrations of the individual samples were calculated using an ATP standard curve. The results are as presented the average of three independent experiments.

### Virulence and FLC efficacy assays in Balb/c mice

Female *BALB/c* mice (Beijing Spaf Biotechnology Co., Ltd) aged 6–8 weeks and weighing 18 ~ 22 g were housed in a standard specific pathogen-free (SPF)-grade animal facility with 12 h of light/12 h of dark conditions, optimal temperature and humidity, filtered water, and appropriate nutritious feed.

Eighty-eight female mice were randomly divided into four groups (SC5314 Group, sdh8*Δ/Δ* group, CP Group, and blank control group) with 22 mice in each group, 5 or 6 mice were housed in a flat-bottomed cage with free access to food and water. Ten of them were used to investigate the survival rate of mice, the other 12 were divided into 2 groups, on Day 2 (6 mice) and day 5 (6 mice) after infection, respectively, the mice were used for kidney fungal burden (right kidney) and PAS staining for pathological section (left kidney). The blank control group was injected with 200 µL sterile saline via the lateral tail vein, and the other groups were injected with the same volume of different strains suspension containing live fungal cells (2.0 × 10^6^ CFU/mL). At the same time, the remaining fungal solution used for injection was diluted to 1 × 10^3^ cells/mL with normal saline, and 100 μL diluted suspension was coated on the YPD solid medium. After being incubated at 30°C for 24 h, the CFU was recorded to verify whether the fungal solution count was correct.

Fungal yeast or hyphae were found in the kidney tissue of with a one-time successful injection of fungal suspension into the tail vein, which was regarded as successful modelling, and mice with injections or suspension leakage were excluded. The animals were closely monitored for 15 d after infection for the development of study endpoint, survival or death. Behavioral activities, food intake, fur, and body weight of the mice were carefully observed and recorded in detail. GraphPad Prism 8.0 statistical software was used for analysis, and survival differences were compared by Log-rank Test.

To evaluate the kidney fungal burden, the kidneys were homogenized on days 2 and 5 post-infection. Homogenates were serially diluted in 0.85% sterile saline and cultured on YPD agar supplemented with 50 µg/mL chloramphenicol. After incubating the plates at 30°C for 24 h, the resulting colonies were counted, and the results were expressed as log_10_ CFU per gram of infected organ, one-way ANOVA was used to test the statistical difference between the three groups. Intact mouse kidneys were collected at the same time points and fixed overnight in 4% paraformaldehyde. The fixed tissues were dehydrated, embedded, sectioned, dewaxed, and subjected to PAS staining. The distribution of *C. albicans* in kidney tissue was observed using an SLIDEVIEW VS200 Research Slide Scanner (Olympus Corporation, Japan).

In the FLC-treated mouse infection model, 66 mice were divided into 3 groups (sdh8*Δ/Δ* group, SC5314 Group and normal saline group) with 22 mice in each group. Similarly, 10 mice were used for survival assay and the other 12 were divided into two groups to collect kidneys for fungal load counting and PAS staining on day 4 and day 9 of treatment, respectively. In this model, mice infected with the same method were intraperitoneally injected with FLC (20 mg/kg) at 24, 48, and 72 h of infection. The mice were monitored daily and differences in survival between groups were compared using the Log-rank test.

The remaining animals were put to death by anaesthesia at the end of the experiment. The animal experiments in this study were approved by the Ethics Committee of Guizhou Medical University (Approval No. 2000455), and the licence number for the use of laboratory animals is SYXK-2018–0001.

### RNA isolation and rt-qPCR analysis

*C. albicans* was activated in YPD medium and expanded culture. To isolate the RNA, *C. albicans* yeast cells were ground and disrupted in liquid nitrogen. An RNA Extraction Kit (Thermo Scientific) was used to isolate the RNA, and the concentration was determined using a NanoDrop2000 ultraviolet spectrophotometer before taking 5 µL of the RNA solution for electrophoresis. Subsequently, an RNA reverse transcription kit (Thermo Scientific) was used for reverse transcription of RNA.

For the quantitative analysis of gene transcription levels, SYBR Green reagent (Thermo Fisher) was used according to the manufacturer’s instructions. The relative transcript levels of target genes were calculated using the 2^−ΔΔCt^ method [22]. β-actin was used as an internal reference gene. The reaction conditions were as follows: 50°C for 2 min, 98°C for 30 sec, 98°C for 15 sec, 60°C for 15 sec, and 72°C for 1 min. Steps 3–5 were repeated 40 times. Dissolution curves were generated using a built-in program of the instrument.

### Cell membrane permeability

PI dye (Sigma, United States) with a final concentration of 10 μg/mL was used to assess the effect of FLC on cell membrane permeability. *C. albicans* cells in the logarithmic growth stage were cultured in YPD liquid medium supplemented with various concentrations of FLC (2 µg/mL, 8 µg/mL, and 16 µg/mL) at a concentration of 2.0 × 10^6^ CFU/mL for 8 h at 30°C with agitation at 200 rpm. Subsequently, a final concentration of 50 µg/mL PI dye was added to each group and incubated for an additional 30 min at 37°C. After incubation, the samples were washed thrice with PBS, followed by detecting on the machine immediately. The ratio of PI-positive cells was rapidly quantified using the CytoFLEX LX flow cytometry (Beckman Coulter, United States), and 10,000 cells were recorded in each sample. The excitation and emission wavelengths used for detection were 535/615 nm.

### Statistical analysis

All data were statistically analysed using GraphPad Prism 8.0 (GraphPad Software, San Diego, CA, USA). Data between two groups were analysed using the Student’s t-test, while data from more than two groups were analysed using a one-way ANOVA. The analysis of survival data was performed using the log-rank test. Data are shown as mean **±** standard deviation (Mean **±** SE), and *p* ≤ 0.05 was considered statistically significant.

## Results

### Orf19.1588 is homologous to SDH8 and localizes in mitochondria

**Figure 1.**
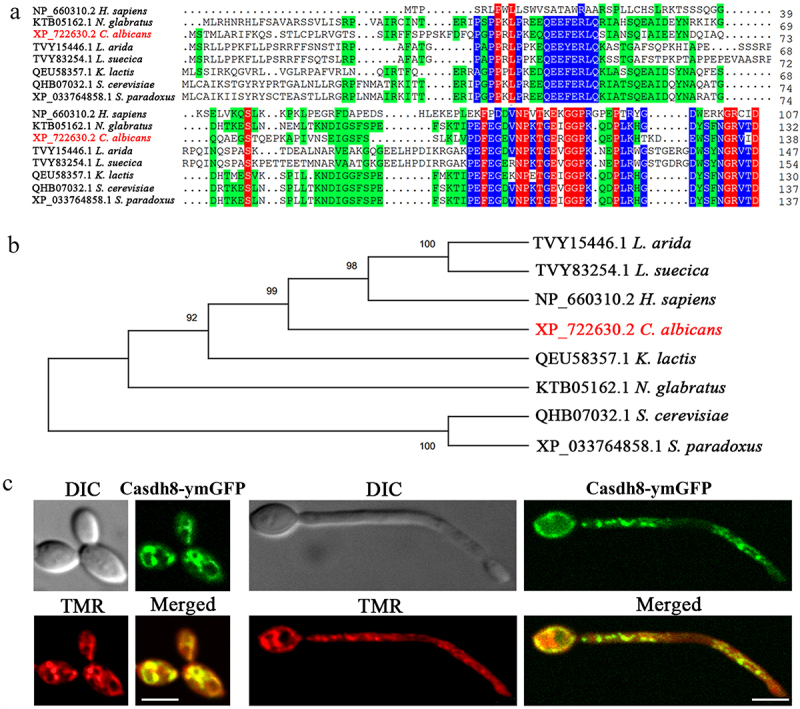
Sequence analysis and intracellular localization of CaSdh8p.

To identify the protein encoded by *orf19.1588*, the amino acid sequence was compared with that of Sdh8p/SDHAF4p in other species using DNAMAN software, revealing an amino acid sequence identity of over 50% (Figure 1a). The SMART: main page (https://smart.embl.de) analysis revealed that the proteins encoded by *orf19.1588* share the same conserved domain-DUF1647 as the Sdh8p in *S. cerevisiae*, suggesting that the two may have similar biological functions. In addition, phylogenetic analysis also demonstrated that the protein encoded by *orf19.1588* was homologous to Sdh8p (Figure 1b). Therefore, the protein encoded by *orf19.1588* in *C. albicans* can be named CaSdh8p. To confirm the subcellular localization of CaSdh8p, *Casdh8-*γm*GFP* strains stained by mitochondrial tracker red (TMR) fluorescent probe were observed using CLSM. The spontaneous green fluorescence of the constructed *Casdh8-*γm*GFP* strain can mostly overlap with the red fluorescence of the TMR staining in both yeast and hyphal cells (Figure 1c), suggesting that *Casdh8* is mainly localised in the mitochondrial organelles of *C. albicans*, and may function in the mitochondria.

(a) Sdh8p sequence alignment. The *SDH8* homologous genes are from *Homo sapiens*, *Nakaseomyces glabratus*, *Candida albicans* SC5314, *Lachnellula arida*, *Lachnellula suecica*, *Kluyveromyces lactis*, *Saccharomyces cerevisiae* and *Saccharomyces paradoxus*. (b) Phylogenic relationships of Sdh8p. The neighbour-joining tree was constructed by MEGA 11, and testing of phylogeny was performed by bootstrap method with 1000 replications. (c) The subcellular localization of CaSdh8p in *C. albicans* was observed by CLSM. Scale bar indicates 5 μm.

### Deletion of SDH8 gene leads to inhibition of hyphal development

**Figure 2.**
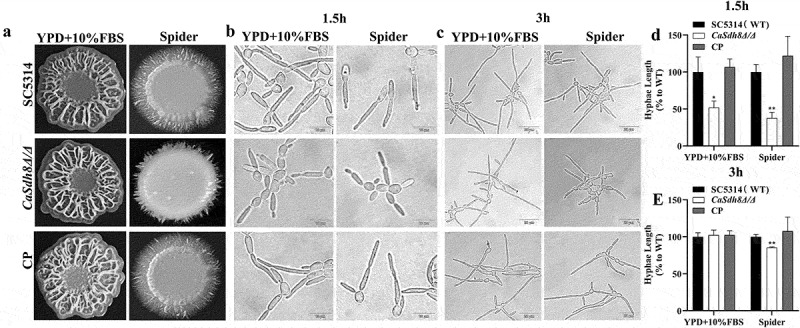
The growth pattern of *C. albicans*.

The *CaSDH8* disrupted strain (*Casdh8Δ/Δ*) and complementary strain (CP) were obtained using CRISPR/Cas9 (Figure S1). To investigate the effect of *CaSDH8* on the ability of *C. albicans* to form hyphae, colony morphology of different strains was observed on solid media. The results showed a wrinkled surface colony morphology in *C. albicans* on YPD + 10% FBS medium, with no significant difference observed between the strains. On Spider medium, the peripheral hyphae of *Casdh8Δ*/*Δ* single colony were less than that of SC5314 and CP strains (Figure 2a). In terms of liquid hyphal development, after 1.5 h of cultivation, *Casdh8Δ*/*Δ* showed an inhibition of hyphal development in YPD + 10% FBS and Spider media, it failed to form normal elongated hyphae or only formed budding yeast or very short pseudohyphae, there was statistical difference in hyphal length between SC5314 and *Casdh8Δ*/*Δ* (Figure 2b–d). After hyphal induction for 3 h, SC5314, *Casdh8Δ*/*Δ* and CP could form elongated hyphal normally, but the hyphal length of *Casdh8Δ*/*Δ* induced by Spider medium was shorter than that of SC5314 and CP (Figure 2c–e). Based on this, we detected the biofilm formation and found that there was no significant difference in biofilm metabolic activity in YPD + 10% FBS medium, but the metabolic activity of *Casdh8Δ*/*Δ* was reduced in Spider medium (Figure S2). Since Spider medium uses mannitol as the sole carbon source, and mannitol belongs to the non-fermentable carbon source, this suggests that deletion of *SDH8* gene may affect *C. albicans*’ utilization of non-fermentative carbon sources, and we will subsequently investigate the utilisation of alternative carbon sources of the *Casdh8Δ*/*Δ*.

(a) Colony of *C. albicans* SC5314, *Casdh8Δ/Δ*, and CP strains incubated at 37°C on solid hyphal induction media of YPD + 10%FBS or Spider for 5 d [58]. (b) Hyphal growth of *C. albicans* incubated at 37°C in liquid hyphal induction media of YPD + 10%FBS or Spider for 1.5 h. (c) Hyphal growth of *C. albicans* incubated at 37°C in liquid hyphal induction media of YPD + 10%FBS or Spider for 3-h Scale bar indicates 10 μm. (d) Length statistics for 1.5 h hyphal development. (e) Length statistics for 3 h hyphal development. Hyphae length was measured by a measurement tool in OlyVIA version 3.3, which was provided by CLSM, and all data have been normalized using the wild type as a reference.

### CaSdh8Δ/Δ has a reduced virulence in mice model of haematogenous disseminated candidiasis

**Figure 3.**
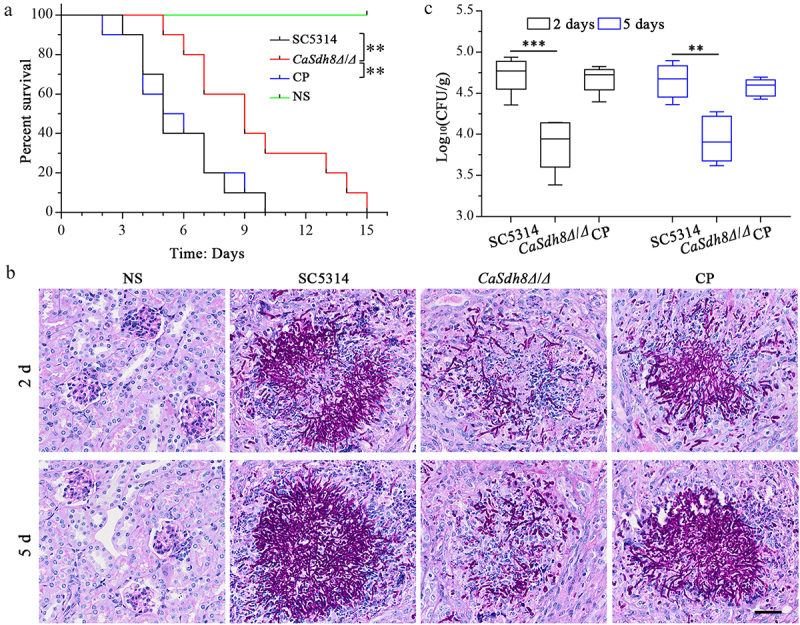
The virulence of *Casdh8Δ/Δ* in mice model of haematogenous disseminated candidiasis.

In order to investigate the influence of *CaSDH8* on the virulence of *C. albicans*, a mouse model of haematogenous disseminated candidiasis was employed to assess the virulence of *Casdh8Δ*/*Δ*. The results revealed that mice infected with *Casdh8Δ*/*Δ* exhibited decreased mortality, and the average survival time increased from 5 to 9 d (Figure 3a). The kidney fungal burden in mice infected with *Casdh8Δ*/*Δ* was significantly lower compared to the SC5314 (WT) and CP strains. Kidney tissues from mice infected with SC5314 and CP strains exhibited significant pathological changes, including tissue heterogeneity, clustering of hyphae, and a high number of neutrophil-based inflammatory cell infiltrates. In contrast, *Casdh8Δ*/*Δ* infected mice showed scattered aggregated hyphae (Figure 3b) and a significantly reduced number of hyphae (Figure 3c). The above results indicate that deletion of *SDH8* resulted in reduced virulence of *C. albicans* in mice.

(a) Survival curves for the mice model of haematogenous disseminated candidiasis infected with *C. albicans* SC5314, *Casdh8Δ/Δ*, and CP. Each group consisted of 10 mice, and 200 μL of 2.0 × 10^6^ CFU/mL was injected. (b) Histopathological PAS staining images of kidney tissue. Scale bar indicates 50 μm. (c) Kidney fungal burden from four groups of mice injected with Normal saline (NS), *C. albicans* SC5314, *Casdh8Δ/Δ*, and CP were obtained at 2 and 5 d after infection. Each group consisted of 6 mice and One-Way ANOVA was used to statistically analyse the results. (*: *p* < 0.05; **: *p* < 0.01; ***: *p* < 0.001; ****: *p* < 0.0001)

### Deletion of CaSDH8 impairs mitochondrial function, resulting in the failure of *C. albicans* to grow on non-fermentative carbon sources

**Figure 4.**
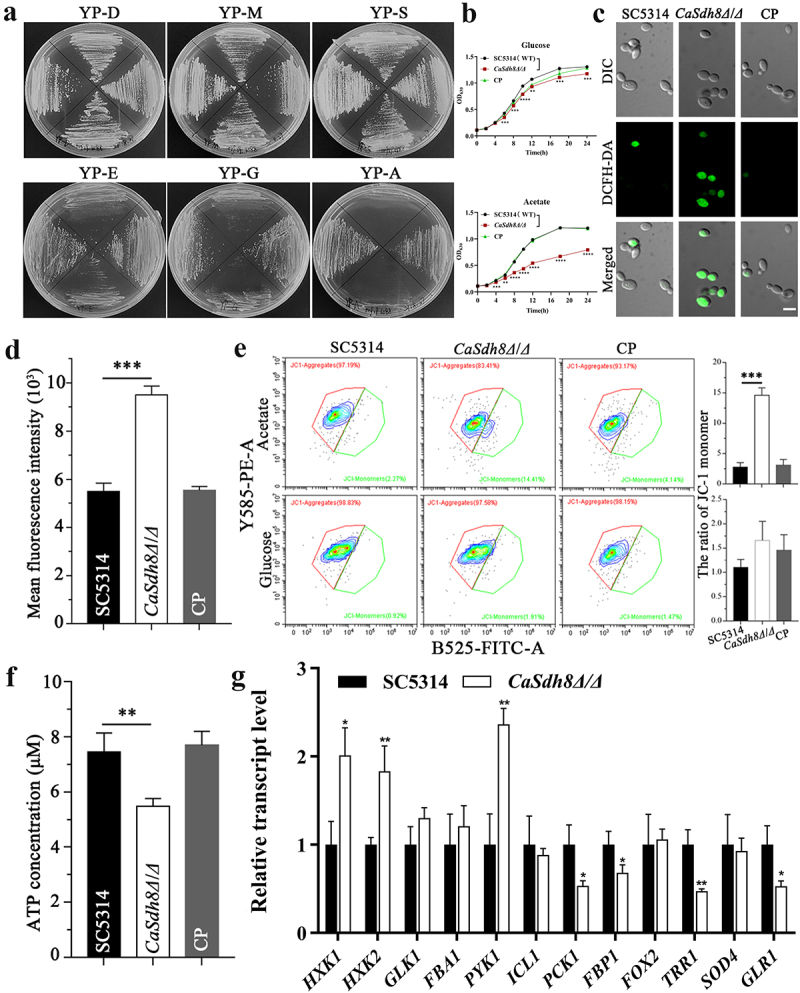
Analysis of mitochondrial function in *Casdh8Δ/Δ*.

The flexibility of *C. albicans* in using alternative carbon sources reflects its mitochondrial respiratory capacity [15]. To investigate the carbon source utilization of *Casdh8Δ/Δ*, we conducted an experiment using three fermentable carbon sources (Dextrose, Maltose, Sucrose) and three non-fermentable carbon sources (Ethanol, Glycerol, Acetate). We observed that *Casdh8Δ/Δ* colonies grew normally on plates containing fermentable carbon sources, similar to SC5314 and CP strains. However, the growth of *Casdh8Δ/Δ* colonies was significantly inhibited on media containing non-fermentable carbon sources, particularly on acetate medium where *Casdh8Δ/Δ* showed almost no growth (Figure 4a). These results indicate that deletion of *CaSDH8* impairs the ability of *C. albicans* to utilize non-fermentable carbon sources.

(a) Growth of SC5314, *Casdh8Δ/Δ* and CP on different solid carbon sources, the images were taken after 24 h of incubation in a 30°C incubator. The left and right side represent SC5314(WT) and CP, respectively, and the upper and lower side represent the *Casdh8Δ/Δ*. (b) Liquid growth assays of SC5314, *Casdh8Δ/Δ* and CP strains under fermentative and non-fermentative carbon source conditions. One-Way ANOVA was used to statistically analyse the results. (c) Images of intracellular ROS in SC5314, *Casdh8Δ/Δ*, and CP cells, which were stained with DCFH-DA dye and captured by CLSM. Scale bar indicates 5 μm. (d) Quantification of intracellular ROS contents. The mean fluorescence intensity of DCFH-DA dye was measured using a multifunctional enzyme marker and statistically analysed using One-Way ANOVA. (e) Mitochondrial membrane potential of SC5314, *Casdh8Δ/Δ*, and CP cells were determined by flow cytometry after staining with JC-1 fluorescent dye. X-axis: B-525-FITC-A, green fluorescence; Y-axis: Y585-PE-A, red fluorescence. A total of 10000 cells were recorded for each sample. Flow cytometry data were analysed using the cytometer’s own software, CytExpert, and statistical difference analyses were performed using the One-Way ANOVA. (f) The intracellular ATP contents of SC5314, *Casdh8Δ/Δ*, and CP strains were detected by chemiluminescence using multifunctional enzyme marker, and statistical difference analyses were performed using the One-Way ANOVA. (g) RT-qPCR analysis of genes related to energy metabolism and oxidative stress in different strains. The relative transcription of genes was expressed as a fold change-(2^−(ΔΔCT)^), and the transcription of genes in SC5314 was used as a reference to calculate the relative transcription of genes in *Casdh8Δ/Δ* and CP. The data were statistically analysed by One-Way ANOVA test. All data are represented as mean ± SD of triplicate sample (*: *p* < 0.05; **: *p* < 0.01; ***: *p* < 0.001; ****: *p* < 0.0001).

We further verified the effect of *CaSDH8* on the liquid growth of *C. albicans*, growth kinetics tests on fermentable and non-fermentable conditions were performed. In the fermentation carbon source (glucose), there was a statistical difference in growth rate between the WT and *Casdh8Δ/Δ* strains from the 6th hour, but the overall growth trend of the *Casdh8Δ/Δ* was only slightly slowed down, which was not obvious to the naked eye. In the non-fermentable carbon source (acetate), the growth rate of WT and *Casdh8Δ/Δ* strains was statistically significant different from the 4th hour, and the growth rate of the *Casdh8Δ/Δ* was significantly slower than the WT (Figure 4b), which was consistent with the trend of the solid carbon source utilization results, and further demonstrates the reduced flexibility of carbon source utilization due to the *SDH8* deletion.

ROS, MMP and ATP are the main indices used to evaluate mitochondrial function. ROS are primarily generated by mitochondria and are known to play a crucial role in fungal cell apoptosis [23]. To assess the ROS levels, we performed microscopic observations using DCFH-DA staining. The results revealed that the ROS level of *Casdh8Δ/Δ* was higher than that of the SC5314 and CP strains (Figure 4c,d), indicating that *SDH8* gene deletion could lead to the accumulation of intracellular ROS on the non-fermentable carbon source in *C. albicans*. Additionally, the accumulation of ROS can cause lipid peroxidation in the mitochondrial membrane, leading to changes in MMP and causing apoptosis [24], so we utilized the JC-1 probe to evaluate the MMP. In normal mitochondria, JC-1 accumulates in the mitochondrial matrix to form polymers, which emit strong red fluorescence; whereas in unhealthy mitochondria, due to the decrease or loss of membrane potential, JC-1 can only exist in the cytoplasm as a monomer, which produces green fluorescence. Therefore, the level of depolarization of the MMP can be evaluated by detecting the ratio of green fluorescence occupying by flow cytometry. By removing cellular debris after selecting the target cell population (Gated cells), subsequent flow cytometry data showed no difference in MMP among the different strains when glucose was used as the carbon source. However, when acetate was used as the carbon source, *Casdh8Δ/Δ* exhibited a higher level of MMP depolarization than the SC5314 and CP strains (Figure 4e). These results suggest that under nutrient-stress conditions, deletion of *CaSDH8* can lead to a decrease in MMP in *C. albicans*, thereby impairing mitochondrial function. Another primary function of the mitochondria is the production of ATP, which provides energy for cellular activities [25]. To investigate changes in ATP concentration in *Casdh8Δ/Δ* cells, we measured the ATP content. The results indicated that the ATP concentration in *Casdh8Δ/Δ* was lower than that in the SC5314 and CP strains (Figure 4f), further supporting the notion that deletion of the *CaSDH8* results in impaired mitochondrial function in *C. albicans*.

Considering the growth impairment of the *CaSDH8* mutant on alternative carbon sources and its repressive effect on mitochondrial activity in *C. albicans*, we further investigated the transcript levels of some genes involved in energy metabolism (glycolysis, glyoxylate cycle, gluconeogenesis, β-oxidation and antioxidants) using RT-qPCR. Our results revealed that the transcript levels of glycolysis-related genes (Hexokinase *HXK1*/*HXK2*, Pyruvate kinase *PYK1*) were significantly increased, whereas the transcript levels of gluconeogenesis-related genes (phosphoenolpyruvate carboxykinase *PCK1*, fructose 1,6-bisphosphatase *FBP1*) and antioxidant-related genes (thioredoxin reductase *TRR1*, glutathione reductase *GLR1*) were down-regulated in *CaSdh8Δ/Δ* compared to SC5314 (Figure 4g). No significant differences were observed in the transcript levels of remaining related genes. These results suggest that *SDH8* deletion may cause more active glycolytic activities by impairing mitochondrial respiration, and the transcript levels of its related genes are therefore increased. At the same time, the inhibition of mitochondrial respiration reduced ATP production and inhibited the anabolism of fungal cells, which led to the down-regulation of the transcription of gluconeogenesis-related genes, while the down-regulation of the antioxidant-related genes was closely related to increased ROS levels.

### CaSDH8 deletion significantly reduces the susceptibility of *C. albicans* to azoles in vitro and in vivo

**Figure 5.**
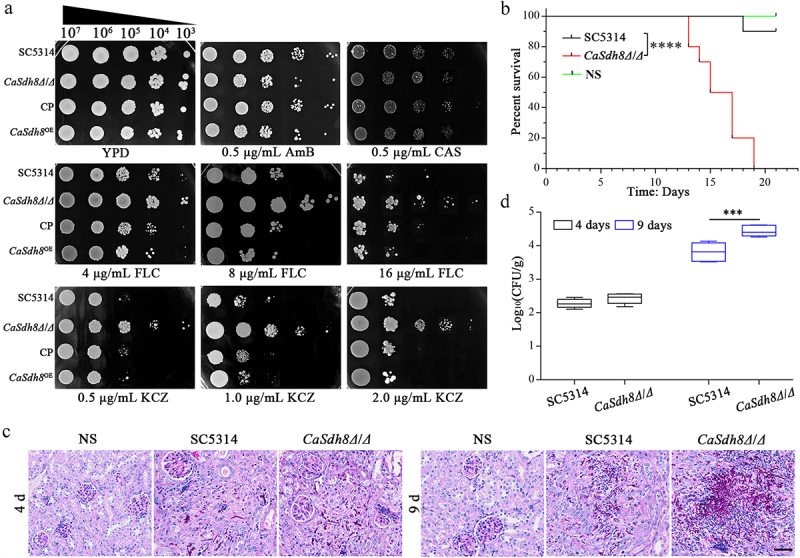
Azole sensitivity of *Casdh8Δ/Δ*.

To determine the minimum inhibitory concentration (MIC) values of the antifungal agents against *C. albicans*, we employed the broth microdilution assay (BDA). The results revealed a significant decrease in susceptibility of *Casdh8Δ/Δ* to fluconazole (FLC) and ketoconazole (KCZ), with MIC values reduced by 64 two-fold concentration gradient compared to SC5314 and CP. The overexpression strain of *CaSDH8* (*Casdh8*^OE^) showed an increased sensitivity to FLC and KCZ compared to SC5314 (Table 2). This indicated that the deletion of *CaSDH8* led to a notable reduction in the efficacy of FLC and KCZ against *C. albicans*. The spot assay test conducted on *Casdh8Δ/Δ* also revealed a decreased sensitivity to various concentrations of FLC and KCZ (Figure 5a). To investigate the relationship between *SDH8* and azole resistance, two clinical strains of azole-resistant *C. albicans* (23–1359 and 23–3192) were collected for further analysis. The amplification of *SDH8* from these strains was successful (Figure S3), and the relative expression of *SDH8* was significantly lower than that of SC5314 (Table S1). These findings suggest that *SDH8* gene may also be related to the azole resistance of clinical azole-resistant strains.

(a) Sensitivities of *Casdh8Δ*/*Δ* to antifungal agents. All strains were grown to the mid-log phase and then spotted on YPD plates in 5-fold dilutions, growth differences were detected after 48 h of incubation. (b) Survival curves for the mice model of haematogenous disseminated candidiasis infected with Normal saline (NS), SC5314, and *Casdh8Δ/Δ*. Each group consisted of 10 mice, and 200 μL of 2.0 × 10^6^ CFU/mL was injected, then the mice model was treated with FLC at a concentration of 20 mg/kg, administered once at the 24th, 48th and 72nd hour after successful infection. (c) Histopathological PAS staining images of kidney. Scale bar indicates 50 μm. (D) Kidney fungal burden of mice infected with different strains after FLC treatment. Kidneys were collected on days 4 and 9 after treatment. Each group consisted of 6 mice and Student’s t-test was used to statistically analyse the results. (*: *p* < 0.05; **: *p* < 0.01; ***: *p* < 0.001; ****: *p* < 0.0001).

**Table 2.** Antifungal susceptibility testing of *C. albicans* strains.

Strains	MIC (μg/mL)			
	AmB	CAS	FLC	KCZ
SC5314	0.5	0.25	4	0.25
*Casdh8Δ/Δ*	0.25	0.125	>512	>32
CP	0.5	0.5	4	0.25
*Casdh8*^OE^	/	/	2	0.125

We confirmed the resistance of *Casdh8Δ/Δ* to FLC *in vitro*. Is this true *in vivo*? To evaluate the therapeutic effect of FLC on mice infected with *Casdh8Δ/Δ*, the mice were infected with different strains and treated with FLC. The results showed that mice infected with *Casdh8Δ/Δ* experienced a rapid increase in mortality by day 13 after treatment, with 100% mortality observed by day 19 after treatment. In contrast, 90% of the mice infected with the SC5314 strain were still alive at that time point (Figure 5b). These findings indicate that deletion of *CaSDH8* significantly reduces the effectiveness of FLC in treating haematogenous disseminated candidiasis in mice. For the assessment of fungal colonization in the kidneys, it was observed that *Casdh8Δ/Δ*-infected mice had a higher presence of aggregated hyphae and inflammatory cell infiltration than SC5314 on the 9th day after treatment (Figure 5c). At the same time, *Casdh8Δ/Δ*-infected mice also carried more fungal load in the kidneys than SC5314 after treatment (Figure 5d). These findings suggest that the reduced sensitivity of *Casdh8Δ/Δ* to azole drugs may result in faster proliferation of fungus *in vivo*, ultimately resulting in more rapid death in *Casdh8Δ/Δ*-infected mice than in SC5314-infected mice following FLC treatment. It was demonstrated that the effectiveness of FLC in treating *Casdh8Δ/Δ*-infected mice was significantly reduced due to the high resistance of the *Casdh8Δ/Δ* to azoles.

### Casdh8Δ/Δ showed decreased FLC-dependent damage to cell membrane permeability and mitochondrial function

**Figure 6.**
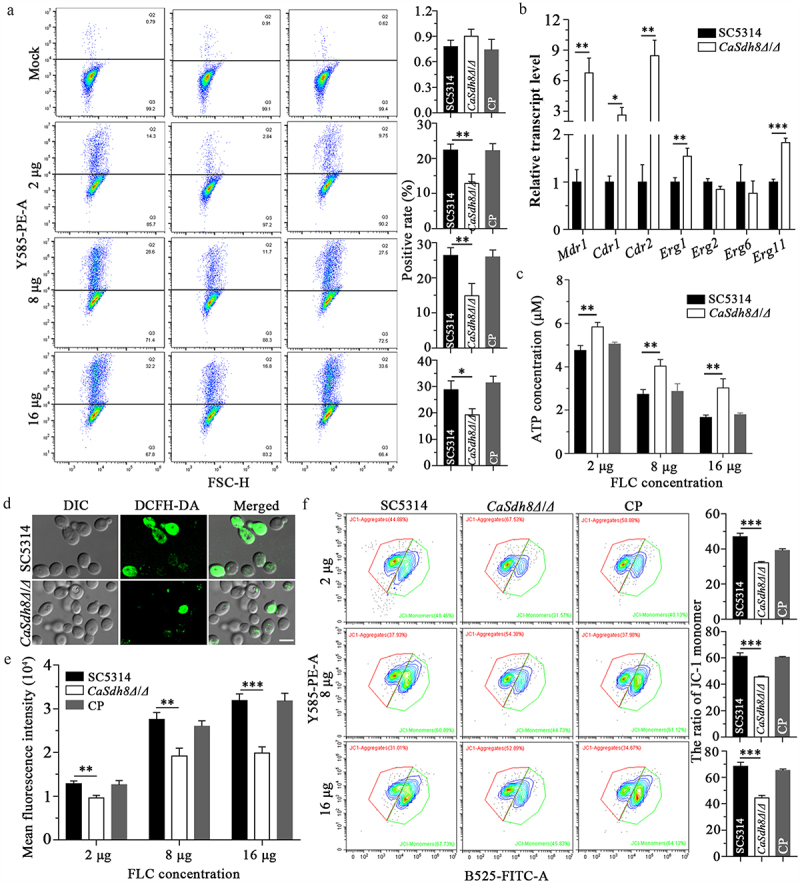
Changes of mitochondrial function, membrane permeability and efflux pump activities in *Casdh8Δ/Δ* under FLC stress.

FLC plays an antifungal role by inhibiting the biosynthesis of ergosterol in the cell membrane, increasing cell membrane permeability and causing the loss of membrane content [7]. To assess the changes of the *Casdh8Δ/Δ* cell membrane permeability under FLC treatment, the cells were stained with PI dye. PI could enter the cell and bind to DNA, thus emitting red fluorescence when the cell membrane permeability was increased. By collecting red fluorescence signals, the ratio of PI-positive showed no significant difference between SC5314 and *Casdh8Δ/Δ*. However, after FLC intervention, the ratio of *Casdh8Δ/Δ* PI-positive cells was reduced compared to that in SC5314 and CP (Figure 6a). This indicates that the deletion of *CaSDH8* does not affect the cell membrane permeability of *C. albicans* but can weaken the damage caused by FLC to the cell membrane. It is well known that ergosterol synthesis and efflux-related genes play a crucial role in azoles [7]. Therefore, we measured the transcription levels of these genes after FLC treatment by RT-qPCR. Interestingly, compared with SC5314, the relative expression of *Mdr1*, *Cdr1*, *Cdr2*, *Erg1*, and *Erg11* in *Casdh8Δ/Δ* cells was significantly increased (Figure 6b). This finding suggests that the reduced susceptibility of *Casdh8Δ/Δ* to azoles can be attributed to the up-regulation of ergosterol synthesis-related genes (*Erg1* and *Erg11*) and efflux-related genes (*Mdr1*, *Cdr1*, and *Cdr2*). This indicates that the deletion of *CaSDH8* does not affect the cell membrane permeability of *C. albicans* but can reduce the damage caused by FLC to the cell membrane.

(a) The ratio between gated cells and PI-positive cells of SC5314, *Casdh8Δ/Δ*, and CP after FLC treatment were detected by flow cytometer. X-axis: Forward Scatter (FSC-H) channel, cell size; Y-axis: Y585-PE-A, PI staining. A total of 10000 cells were recorded for each sample and CytExpert software was used to analyse the ratio of flow cytometry data. The One-Way ANOVA was used for statistical difference analysis. (b) RT-qPCR analysis of the transcript levels of efflux pump and ergosterol synthesis-related genes in SC5314, *Casdh8Δ/Δ*, and CP strains. (c) The content of ATP in SC5314, *Casdh8Δ/Δ*, and CP cells treated with FLC was detected by chemiluminescence using multifunctional enzyme marker, and student’t test was used for statistical analysis. (d) Images of intracellular ROS after 8 μg/mL FLC treatment in SC5314, *Casdh8Δ/Δ*, and CP cells, which were stained with DCFH-DA dye and captured by CLSM. Scale bar indicates 5 μm. (e) Intracellular ROS content after 8 μg/mL FLC treatment of the strains. The mean fluorescence intensity of DCFH-DA dye was measured using a multifunctional enzyme marker and statistically analysed using One-Way ANOVA. (f) Mitochondrial membrane potential of SC5314, *Casdh8Δ/Δ*, and CP cells after FLC treatment were determined by flow cytometry after staining JC-1 fluorescent dye. X-axis: B-525-FITC-A, green fluorescence; Y-axis: Y585-PE-A, red fluorescence. A total of 10000 cells were recorded for each sample and CytExpert software was used to analyse the ratio of flow cytometry data. The One-Way ANOVA was used for statistical difference analysis. All data are represented as mean ± SD of triplicate sample (*: *p* < 0.05; **: *p* < 0.01; ***: *p* < 0.001; ****: *p* < 0.0001).

In addition, FLC can cause mitochondrial dysfunction in *C. albicans* by inducing ROS accumulation and increasing the depolarization level of the MMP, leading to cell apoptosis, thus exerting antifungal activity [26]. Given the essential role of mitochondria in energy production, we measured the intracellular ATP levels in *C. albicans*. These findings demonstrated that as the concentration of FLC increased, the ATP levels in *Casdh8Δ/Δ* decreased. Interestingly, we observed that ATP levels in *Casdh8Δ/Δ* were higher than those in the SC5314 and CP strains in the presence of FLC (Figure 6c). Importantly, ROS accumulation in *Casdh8Δ/Δ* cells was lower than that observed in SC5314 and CP strains (Figure 6d,e). These findings suggest that deletion of *CaSDH8* could mitigate oxidative damage to *C. albicans* by reducing intracellular ROS accumulation under FLC treatment. Additionally, depolarization of MMP in *C. albicans* increased progressively with increasing FLC concentration. Notably, MMP depolarization in *Casdh8Δ/Δ* cells was lower than that in SC5314 and CP cells when subjected to FLC treatment (Figure 6f). These observations suggest that, under FLC stress, the deletion of *CaSDH8* may mitigate mitochondrial damage by reducing the accumulation of ROS and the loss of MMP and ATP, thereby maintaining a more stable state of energy metabolism in *C. albicans*. This might also contribute to the resistance of *Casdh8Δ/Δ* to azoles.

## Discussion

In our study, we found that the *SDH8* gene, localized in the mitochondria, has a significant impact on the growth of alternative carbon sources and is necessary for the complete virulence and susceptibility response to azoles in *C. albicans*. SDH serves as a component of mitochondrial complex II (CII), which plays a crucial role in transferring electrons from succinate to ubiquinone for energy release in the mitochondrial oxidative respiratory chain [13]. Several studies have suggested that CII is essential for morphological transformation, and the TTFA inhibitor of CII completely inhibits hyphal formation in *C. albicans* [27]. *CaSDH8* is one of the assembly factors of SDH, and its absence may affect the activity of the CII complex, consequently leading to a lag in hyphal development. Hyphal formation in *C. albicans* involves a variety of complex mechanisms, and hyphal cells respond differently to different environmental stimuli. *SDH8* mutants only showed a delay in the initiation of hyphal development, which may be due to the regulation of other compensatory mechanisms.

In this study, the deletion of *CaSDH8* resulted in decreased virulence in the *CaSDH8* knockout strain. The virulence of *C. albicans* relied heavily on the integrity of mitochondrial function, and any null mutation that affects mitochondrial function can lead to a defect in virulence [28–32]. Mitochondria are the primary sites of intracellular ROS accumulation, and an excessive increase in ROS can lead to mitochondrial dysfunction, oxidative damage, and cellular dysfunction [33]. MMP is a direct indicator of mitochondrial function and reflects the aerobic metabolism level of fungi, which is necessary for hyphal formation [34]. A decrease in MMP reduces the production of ATP and the supply of energy to cells, which affects normal cellular metabolism [35,36]. By measuring ROS, MMP, and ATP levels, we observed impaired mitochondrial function in *CaSDH8Δ/Δ* under non-fermentation carbon source conditions. When *C. albicans* colonizes a host, it is challenged by a nutritive environment in which there is a lack of glucose [37]. We speculated that in this case, the mitochondrial function of *CaSDH8Δ/Δ* was inhibited, which may be related to the decreased fungal load in the kidney of the mouse colonization site and reduced virulence in mice.

In addition, the ability of *C. albicans* to utilize alternative carbon sources is indicative of its mitochondrial respiratory capacity [15]. In *C. albicans*, mitochondrial mutants with impaired energy metabolism exhibit an inability to grow and form small colonies on non-fermentable carbon sources [38], primarily due to their lower cell division rates compared to normal cells [39]. It has been reported that the *SDH8* null mutant in *S. cerevisiae* exhibits normal growth in glucose media but poor or no growth in glycerol and acetate media, which serves as a marker for SDH activity and suggests a deficiency in mitochondrial respiration [15]. These findings are consistent with our results, the growth rate and hyphal elongation of the *CaSDH8Δ/Δ* on solid and liquid non-fermenting carbon sources was inhibited after *CaSDH8* deletion. In glucose conditions, although the absence of *CaSDH8* does not significantly alter the overall growth pattern of *C. albicans*, it results in a slowdown of growth and reproduction, a phenomenon observed in *SDH8* mutant of *S. cerevisiae* [15]. On this basis, we also found that under fermentation carbon source, the relative transcript levels of partial genes related to glycolytic pathway (*HXK1*/*HXK2*/*PYK1*) were up-regulated after *SDH8* deletion, which may be due to the fact of that the deletion of *SDH8* caused diminished activity of SDH, thus inhibited mitochondrial respiration through the disruption of succinate oxidative respiratory chain (complex II), and feedback enhanced the glycolytic activity. Meanwhile, due to the weakening of mitochondrial respiration, ATP production was reduced, which could not provide sufficient energy for the anabolic metabolism such as gluconeogenesis in the fungal cells, and caused the transcript levels of gluconeogenesis-related genes (*PCK1*/*FBP1*) to be down-regulated as well.

In addition to changes in genes related to energy metabolism, antioxidant-related genes are also critical for the survival and regulation of *C. albicans*. *C. albicans* has various pathways to counteract oxidative damage, such as the induction of the glutathione (Glr1p) system and the thioredoxin system (Trr1p), as well as repair of oxidative damage to protein thiol groups in response to reactive oxygen species [40,41]. In our study, we observed a decrease in the relative transcription of antioxidant-related genes *TRR1* and *GLR1*, as well as an accumulation of ROS in *Casdh8Δ/Δ* cells. The decrease of these genes can cause dysregulation of redox homoeostasis and an inability to properly scavenge intracellular ROS, leading to increased oxidative damage in *C. albicans*. In the context of host–pathogen interactions, the ability of *C. albicans* to withstand high levels of ROS is crucial, as the capacity for antioxidant damage is closely linked to its virulence properties, including morphological transformation and metabolic flexibility [42,43]. Based on these findings, we hypothesized that the reduced expression of these genes may result in a diminished capacity for antioxidant damage in *C. albicans*, ultimately affecting its virulence.

Besides, *Casdh8Δ/Δ* showed more prominent azole resistance. Our results revealed a significant decrease in susceptibility of *Casdh8Δ/Δ* to FLC and KCZ, with MIC values reduced by 64 two-fold concentration gradient. *In vivo* efficacy experiments in mice also showed that the *CaSDH8* null mutation was less effective in treating mice due to reduced sensitivity to FLC, which provided a new clue for studying the mechanism of fungal resistance to azole drugs *in vivo*.

Clinical studies have shown that the resistance of azoles to pathogenic fungi is mainly related to changes in ergosterol biosynthesis and overexpression of efflux pump membrane transporters [44]. Among them, the ergosterol synthesis can directly affect the permeability of fungal cell membrane, and clinical azole-resistant strains usually overexpress ERG genes related to ergosterol synthesis [45,46]. Besides, increased resistance is associated with increased efflux pump activity, which results in a reduced intracellular antifungal drug accumulation [47]. Genes related to efflux pump mainly consist *CDR1, CDR2*, and *MDR1*, which frequently show increased expression and efflux activity in azole-resistant strains [48,49]. Our results showed that the absence of *CaSDH8* did not obviously affect the cell membrane permeability of *C. albicans*, but could significantly weaken the destruction of the cell membrane by FLC, while up-regulating the transcript levels of *Erg1* and *Erg11* genes related to ergosterol synthesis and *CDR1*, *CDR2*, *MDR1* genes related to efflux pumps. Combined with our findings, the reduced susceptibility of the *Casdh8Δ/Δ* to FLC was mainly due to weakened membrane damage and upregulated transcript activity of genes related to ergosterol synthesis and efflux pump.

In addition, our data showed that *Casdh8Δ/Δ* had a lower accumulation of intracellular ROS and loss of MMP and ATP than WT after FLC treatment. Therefore, we believe that the absence of *CaSDH8* lessens the damage to mitochondrial function in FLC to *C. albicans*, thereby reducing the sensitivity of *C. albicans* to FLC. Yan Lan [50,51] reported that one of the mechanisms by which drug resistance in *C. albicans* arises is when the classical oxidative respiratory pathway is inhibited or blocked in favour of alternate oxidation as the primary electron transport pathway. The elevated ROS level in *C. albicans* cells during drug action is reduced, thus reducing the sensitivity of the strain to azoles. One of the fungal self-defence mechanisms is the inducible production of alternate respiration, which is typically a stress response to physiological conditions or changes in the external environment [52]. Thus, loss of *CaSDH8* is likely to induce alternate oxidative respiration and lead to azole resistance. In addition, the mitochondrial respiratory function of *C. albicans* was found to be significantly reduced in drug-resistant offspring, mainly in the form of lower MMP and intracellular ATP levels in resistant offspring than in sensitive parent [50,51]. Earlier studies in *S. cerevisiae* have revealed that the inhibitory effect of FLC requires the presence of well-functioning mitochondria [53,54]. Blocking mitochondrial respiratory chain complex IV can lead to the development of high resistance to azoles in *C. glabrata* [55–57]. These studies showed that mitochondrial defects or blocked mitochondrial respiratory function in both *S. cerevisiae* and *C. glabrata* can lead to reduced susceptibility of these strains to azoles, further demonstrating that mitochondrial function plays an essential role in the development of drug resistance in *C. albicans*. This is also consistent with our finding that *CaSDH*8 deletion disrupts mitochondrial function and leads to resistance to azole drugs.

In summary, the pathogenesis of *C. albicans* infection is complex and involves multiple factors. Our study provides the first evidence that the loss of *CaSDH8* inhibits the growth of alternative carbon sources and virulence of *C. albicans* by impairing mitochondrial function. Furthermore, we uncovered a novel function for *CaSDH8* in susceptibility to azoles. Deletion of *CaSDH8* weakened the damage to the cell membrane and mass disruption of mitochondrial function in *C. albicans* under FLC treatment compared to the WT strain, while increasing the expression level of efflux pump-related genes, thus showing a significantly reduced sensitivity to azole drugs. This obvious reduction in susceptibility to azoles due to *CaSDH8* deletion leads to a significant decrease in the effectiveness of fluconazole in treating mouse models infected with the deletion strain, which has significant implications for the study of fungal mechanisms of azole resistance and the screening of antifungal drug targets.

## Supplementary Material

Supplemental Material

Table_S4.docx

Table_S2.docx

Table_S3.docx

Table_S1.docx

## Data Availability

The data from this study are available in Science Data Bank (DOI: https://doi.org/10.57760/sciencedb.08468).
