# Supplementary figures and images for: Unveiling the roles of CaSDH8 in Candida albicans: Implications for virulence and azole resistance
Source: Virulence. 2024 Oct 15;15(1):2405000. doi: 10.1080/21505594.2024.2405000 (PMC11485852; doi:10.1080/21505594.2024.2405000)

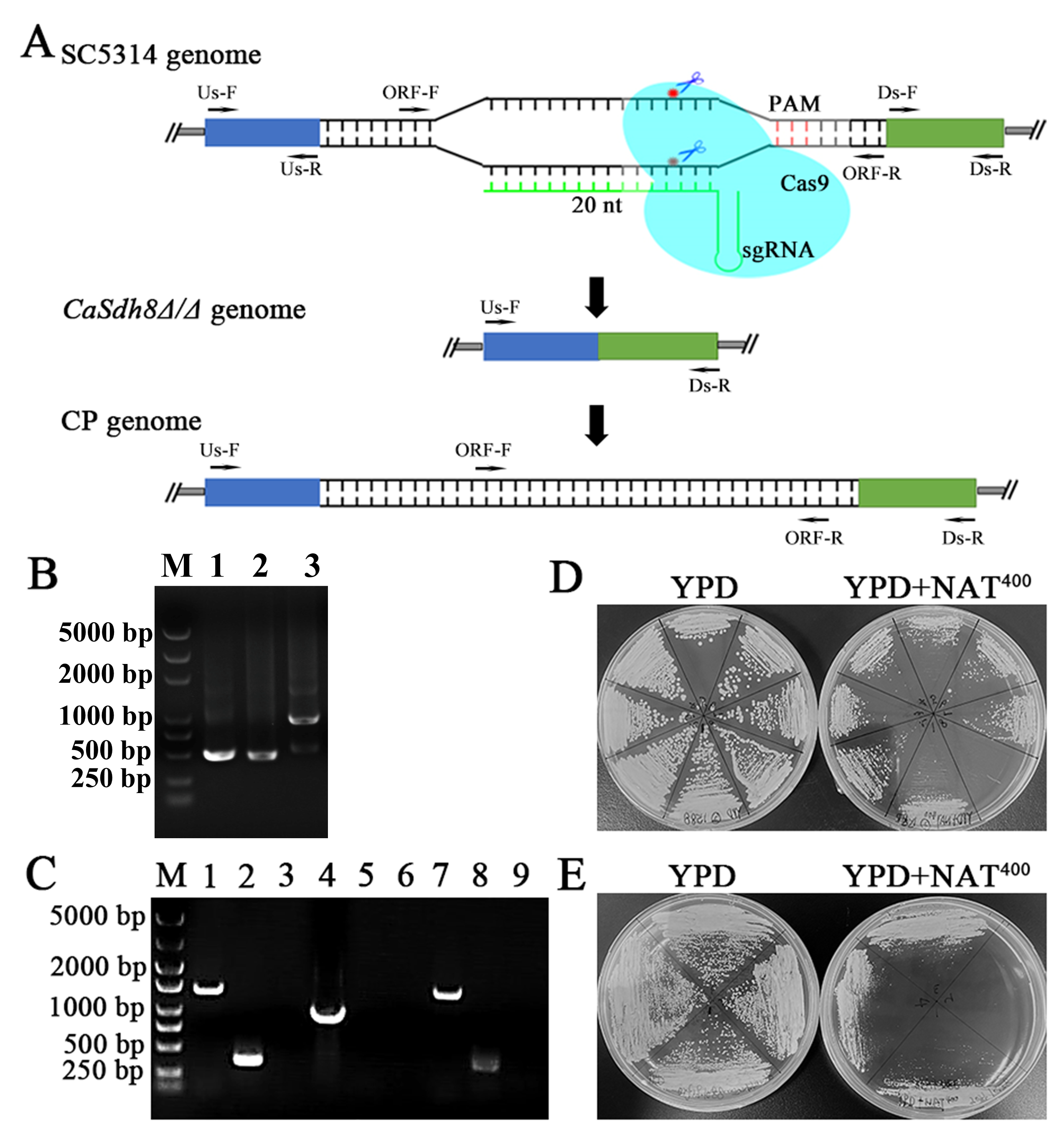

Supplement: Supplemental Material [file KVIR_A_2405000_SM0432.zip › S1_Fig.tif]

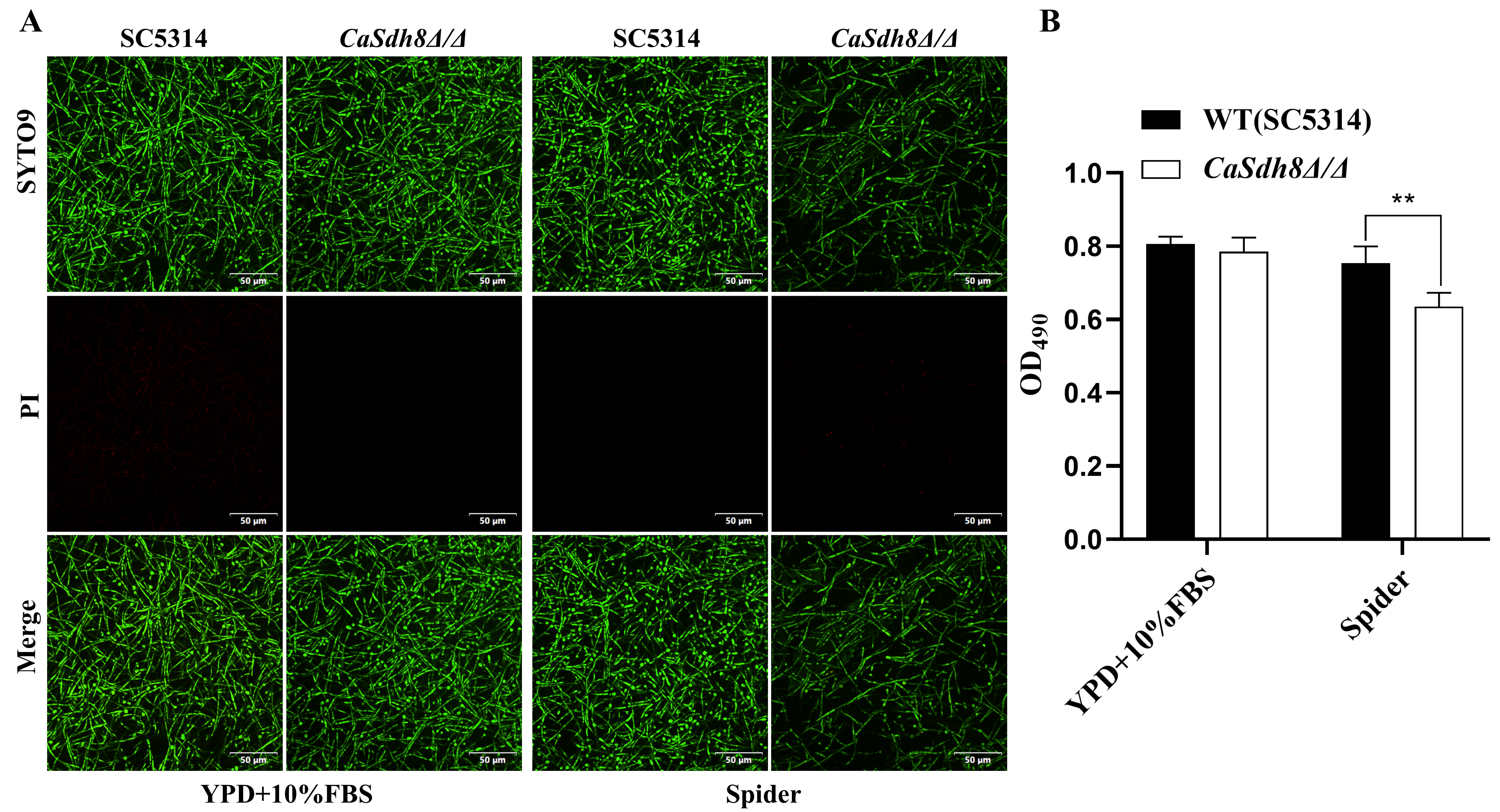

Supplement: Supplemental Material [file KVIR_A_2405000_SM0432.zip › S2_Fig.tif]

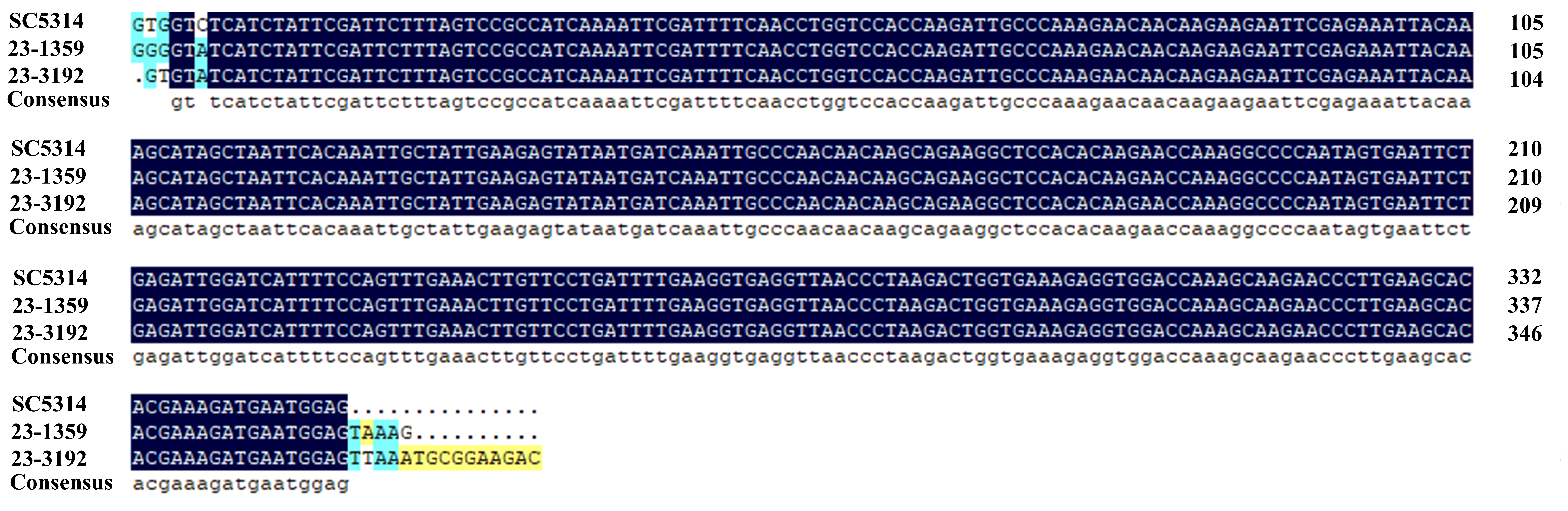

Supplement: Supplemental Material [file KVIR_A_2405000_SM0432.zip › S3_Fig.tif]
