# Supplementary material for: Unveiling the roles of CaSDH8 in Candida albicans: Implications for virulence and azole resistance
Source: Virulence. 2024 Oct 15;15(1):2405000. doi: 10.1080/21505594.2024.2405000 (PMC11485852; doi:10.1080/21505594.2024.2405000)
Supplement: Table_S4.docx [file KVIR_A_2405000_SM0169.docx]

**Table S4 Sequences of qPCR primers for target genes**

| Gene | Primer sequence（5’ to 3’） | Lengh |
| --- | --- | --- |
| β-actin | ACCGAAGCTCCAATGAATCC | 20 |
| β-actin | CCGGTGGTTCTACCAGAAGAG | 21 |
| *TRR1_F* | AAGGTGTGTTTGCAGCTGGT | 20 |
| *TRR1_R* | TCCAAAGCAGCCATACATCCA | 21 |
| *SOD4_F* | GCTACCTTGAGAAGCACT | 18 |
| *SOD4_R* | GCTTACATTGGTGGGTTG | 18 |
| *GLR1_F* | CCAGCACAAATGATCTTG | 18 |
| *GLR1_R* | CCAGAAGCTGGTTCTATT | 18 |
| *HXK1_F* | ATGTGGGGTCGGTCTTGAAC | 20 |
| *HXK1_R* | GCACCTATGGCAGCACCTAT | 20 |
| *HXK2_F* | TGCCATTGACTTGGGTGGAA | 20 |
| *HXK2_R* | CAGGGTGGCAGTTCTCATGT | 20 |
| *GLK1_F* | AACCAACAAGGAGGAACGTG | 20 |
| *GLK1_R* | GCAATTGGGATCGCAGACAA | 20 |
| *FBA1_F* | TGCCAGAGACAACAAGGCTC | 20 |
| *FBA1_R* | GGCAGCAATTGAACCAGCAA | 20 |
| *PYK1_F* | AAAAGGCCATTGCCTACCCA | 20 |
| *PYK1_R* | CGGCAACAGCACAAGTTTCA | 20 |
| *FOX2_F* | CACATACCGTGCAAGATT | 18 |
| *FOX2_R* | CGCTGAACGTAGATCATT | 18 |
| *ICL_F* | TGTTGCCAGAGCCAGAGAAG | 20 |
| *ICL_R* | GGCATAATCTGGCAAAGCGG | 20 |
| *PCK1_F* | GTGATGCTTCCGGTGTGTTG | 20 |
| *PCK1_R* | GTTGACCGAAACATGCGGAG | 20 |
| *FBP1_F* | ATTGGGCTGATGGTTGCTGA | 20 |
| *FBP1_R* | AACAAGGCCATGGGGAAACA | 20 |
| *MDR1_F* | CCACATCAGCAACACTTG | 18 |
| *MDR1_R* | TATGGTGTTGGCCCATTG | 18 |
| *CDR1_F* | CTAAGATGTCGTCGCAAG | 18 |
| *CDR1_R* | CTGCTGACGAGTCATCTT | 18 |
| *CDR2_F* | CACGTCTTTGTCGCAACA | 18 |
| *CDR2_R* | TATGTCGGACATGTGGCT | 18 |
| *ERG1_F* | GACCGAATAGAAGGCAACAC | 20 |
| *ERG1_R* | GGACGCTGTTATCAACACAT | 20 |
| *ERG2_F* | GCCTTGAGCCAATTCTAAGG | 20 |
| *ERG2_R* | CTGGTGAACAAAGAGCAGCT | 20 |
| *ERG6_F* | CCATCACCGACTTCAATACC | 20 |
| *ERG6_R* | GCTGTTTATGCCATTGAAGC | 20 |
| *ERG11_F* | CACGTCTCCAATAATGAGGT | 20 |
| *ERG11_R* | CATGGGGTTGCCAATGTTAT | 20 |
| *SDH8_F* | GTCTACCATGTTAGCAAGAA | 20 |
| *SDH8_R* | GTTCTTTGGGCAATCTTGGT | 20 |
