# Supplementary material for: Unveiling the roles of CaSDH8 in Candida albicans: Implications for virulence and azole resistance
Source: Virulence. 2024 Oct 15;15(1):2405000. doi: 10.1080/21505594.2024.2405000 (PMC11485852; doi:10.1080/21505594.2024.2405000)
Supplement: Table_S2.docx [file KVIR_A_2405000_SM0167.docx]

**Table S2 Target gRNA sequences**

| Gene | RNA sequence（5’ to 3’） | PAM sequence | Reference |
| --- | --- | --- | --- |
| *SDH8* | GGTGAGGTTAACCCTAAGAC | TGG | This study |
| NeutL5 | CATCGTCCTTCCTAAACAAG | TGG | This study |
