# Supplementary material for: Unveiling the roles of CaSDH8 in Candida albicans: Implications for virulence and azole resistance
Source: Virulence. 2024 Oct 15;15(1):2405000. doi: 10.1080/21505594.2024.2405000 (PMC11485852; doi:10.1080/21505594.2024.2405000)
Supplement: Table_S3.docx [file KVIR_A_2405000_SM0166.docx]

**Table S3 Upstream, downstream, and ORF primer sequences of target genes**

| Primer | Primer sequence（5’ to 3’） | Length | |
| --- | --- | --- | --- |
| *SDH8_Up_F* | ggtgaggttaaccctaagac | 20 |  |
| *SDH8_Up_R* | CCTCATGTCGAGCACTCGTCTCGttaataaattgggtttagagac | 45 |  |
| *SDH8_Down_F* | CGAGACGAGTGCTCGACATGAGGgttatatcattagtgtaaggat | 45 |  |
| *SDH8_Down_R* | ggcatctcacaaaaatagcgat | 22 |  |
| *SDH8_ORF_F* | caatcttccactttatgccctt | 21 |  |
| *SDH8_ORF_F* | ctctcccattaaaactccattc | 22 |  |
| *SDH8_Up_F1* | GGACAGCAAATGGGTCGCGGATCCggtacccgaatcgtattgttttatta | 50 |  |
| *SDH8_ Up_R1* | GTGAATAATTCTTCACCTTTAGACATGTCGACgaaatcaataactctcccattaa | 55 |  |
| *SDH8_Down_F1* | GAACGTTATTTATATTTCAAATTAAGCTTgttatatcattagtgtaagg | 49 |  |
| *SDH8_Down_R1* | TCAGTGGTGGTGGTGGTGGTGCTCGAGttctttcttgttttatgtcaatgat | 52 |  |
| *γmGFP_F1* | GAGTTATTGATTTCGTCGACatgtctaaaggtgaagaattattcac | 46 |  |
| *γmGFP_R1* | ctaatgatataacAAGCTTAATTTGAAATATAAATAACGTTCTT | 44 |  |
| *HR_(SDH8-GFP )__F* | ggtacccgaatcgtattgttttatta | 26 |  |
| *HR_(SDH8-GFP )__R* | ttctttcttgttttatgtcaatgat | 25 |  |
| *SDH8_C_F* | actaacacaatcaacaaatctacctt | 26 |  |
| *SDH8_C_R* | ctctcccattaaaactccattcatct | 26 |  |
| *GFP_C_F1* | agaagatggtaacattttaggtcac | 25 |  |
| *GFP_C_R1* | tgcgtctggatcatcattgtactc | 24 |  |
| *SDH8_Up_F2* | GGACAGCAAATGGGTCGCGGATCCggtacccgaatcgtattgttttatta | 50 |  |
| *SDH8_Up_R2* | AGTTGAAGATAGTTGCTACATGAGCTcttaataaattgggtttagagac | 49 |  |
| *ENO1_Pro_F* | CCAATTTATTAAGAGCTCatgtagcaactatcttcaact | 39 |  |
| *ENO1_Pro_R* | CATGGTAGACATGTCGACgttgtaatattcctgaattatc | 40 |  |
| *SDH8_ORF_F1* | TCAGGAATATTACAACGTCGACatgtctaccatgttagcaagaatat | 47 |  |
| *SDH8_ORF_R1* | TCAGTGGTGGTGGTGGTGGTGCTCGAGttctttcttgttttatgtcaatgat | 47 |  |
| *HR_(Pro-SDH8 )__F* | ggtacccgaatcgtattgttttatta | 26 |  |
| *HR_(Pro-SDH8 )__R* | ttctttcttgttttatgtcaatgat | 25 |  |
| *SDH8_C_F1* | actaacacaatcaacaaatctacctt | 26 |  |
| *SDH8_C_R1* | ctctcccattaaaactccattcatct | 26 |  |
