# Supplementary material for: Unveiling the roles of CaSDH8 in Candida albicans: Implications for virulence and azole resistance
Source: Virulence. 2024 Oct 15;15(1):2405000. doi: 10.1080/21505594.2024.2405000 (PMC11485852; doi:10.1080/21505594.2024.2405000)
Supplement: Table_S1.docx [file KVIR_A_2405000_SM0165.docx]

**Table S1 Relative expression of *Sdh8* in each strain**

| Strains | Relative expression of *Sdh8* gene |
| --- | --- |
| SC5314 | 1±0.1179 |
| *CaSdh8*^OE^ | 56.16±7.311** |
| 23-1359 | 0.001491±0.0001198** |
| 23-3192 | 0.000463±5.973e-005** |
